# Printed Circuit Boards: The Layers’ Functions for Electronic and Biomedical Engineering

**DOI:** 10.3390/mi13030460

**Published:** 2022-03-17

**Authors:** Francisco Perdigones, José Manuel Quero

**Affiliations:** Electronic Engineering Department, University of Seville, 41092 Sevilla, Spain; quero@us.es

**Keywords:** Printed Circuit Board (PCB), biomedical, electronic, engineering

## Abstract

This paper describes the fabrication opportunities that Printed Circuit Boards (PCBs) offer for electronic and biomedical engineering. Historically, PCB substrates have been used to support the components of the electronic devices, linking them using copper lines, and providing input and output pads to connect the rest of the system. In addition, this kind of substrate is an emerging material for biomedical engineering thanks to its many interesting characteristics, such as its commercial availability at a low cost with very good tolerance and versatility, due to its multilayer characteristics; that is, the possibility of using several metals and substrate layers. The alternative uses of copper, gold, Flame Retardant 4 (FR4) and silver layers, together with the use of vias, solder masks and a rigid and flexible substrate, are noted. Among other uses, these characteristics have been using to develop many sensors, biosensors and actuators, and PCB-based lab-on chips; for example, deoxyribonucleic acid (DNA) amplification devices for Polymerase Chain Reaction (PCR). In addition, several applications of these devices are going to be noted in this paper, and two tables summarizing the layers’ functions are included in the discussion: the first one for metallic layers, and the second one for the vias, solder mask, flexible and rigid substrate functions.

## 1. Introduction

The challenge of interconnection between electrical and electronic components led the development of several methods, which can be used to perform this task in a reliable and inexpensive manner. This took place during the end of the 19th century and at the beginning of the 20th century [1,2]. Previously, the interconnection was performed manually using wires. These methods provide the base and origin of the current Printed Circuit Board (PCB).

It is difficult to establish the beginning and inventor of the first Printed Circuit Board. This is because it has the characteristics of several inventions. The first technique used to perform something similar to a double-side PCB was developed by Baynes in 1888 [3] using exposing and etching techniques. However, the functionality of the result was purely ornamental, far from an electronic interconnection. Several years later, Hanson (1903) [4] developed the patent “Electric cable”, where an insulating substrate with integrated wires was intended for electrical purposes. This concept is similar to the current single and multilayer PCBs. After that, in 1913, Arthur Berry presented several patents [5] “Improvements in or relating to Electric Heating Apparatus”, where the etching technique is used for electro-thermal applications: for fabricating heaters. These thermal devices could be considered one of the first integrated actuators on an insulating substrate. Then, in 1925, Charles Ducas [6] developed a panel containing the metalized lines and subjected it to electroplating to deposit the additional metal. About ten years later, Paul Eisner [7] defined a method to fabricate a stack of metal and insulating materials, which could be considered the first PCB. Finally, Hutters [8] patented the “electrical component mounting device” using hole technology in 1955, and Gabrick [9] defined a composition of the solder mask.

After these developments, a huge quantity of PCBs were fabricated for many different applications. At present, the majority of devices include an electronic part, and this part is made of Printed Circuit Boards; for example, devices for medical applications, such as defibrillators, anesthesia machines, electrocardiogram machines, electrosurgical units and pacemakers to name a few. The devices for industrial equipment also include PCBs; for example, power supplies, computer numerical control (CNC) machines, power inverters or solar power cogeneration devices, to name a few. In addition, automotive, aerospace/satellital and maritime devices include PCBs as electronic substrates for the circuits; for instance, navigation systems, microcontrollers, conditioning electronic circuits for sensors and actuators, and communication equipment. Finally, the consumer electronics, such as videogames, electrical appliances, personal computers and smart phones, form a representative example.

The use of PCB substrates for these applications is well-known and well-established. This has led the creation of many commercial companies, which offer a rapid and low-cost fabrication of PCBs with very good characteristics. These advantages, that is, their low cost and precise dimensions, together with the characteristics of the PCB, made this substrate an interesting material to perform applications for which the PCB was not intended. Regarding this, microelectromechanical systems (MEMS), which typically and historically used silicon as base material, adopted the Printed Circuit Board as an alternative material due to its advantage of having one or more copper layers to fabricate microchannels, sensors or actuators, and the ability to include microfluidics and electronics in the same substrate at a low cost [10,11,12,13,14,15]. These devices were named PCB-MEMS, and formed the origin of much more developments thanks to the application of lab-on-a-chip (LoC) devices.

Lab-on-a-chips are biomedical, biochemical or chemical devices, including several laboratory functions in a substrate with the dimensions of a credit card, or even smaller. They could include microheating, micropumping, temperature sensors, biological parameter sensors, micromixing and different detection systems. The first LoC materials were silicon and glass. These materials are expensive when considering the typical dimensions of a lab-on-a-chip. For that reason, these materials are going to be replaced by polymers, such as polydimethylsiloxane (PDMS) and SU-8 for prototyping purposes, and thermoplastics for industrial applications. The use of these polymeric materials implies a lack of electrical connections to link the device with the control system, and to integrate sensors and actuators with ease. In this respect, PCBs provide functionalities to solve these problems. Furthermore, the secondary functions of PCB are interesting when looking at the fabrication and performance of the devices.

This paper describes the functionalities of the Printed Circuit Boards layers for different applications to the typical one. The alternative uses of the copper layers, the solder mask, the vias and the PCB substrate are described. Representative devices fabricated using these alternative technologies are noted, emphasizing biomedical and electronic engineering. These technologies will be analyzed in the Section 6 and a conclusion is given in the Section 7. It is important to highlight that this paper is not a comprehensive review of PCB-based devices, but a description of devices that use PCBs as a structural and functional material. More importantly, this paper shows the possibilities that the different layers of PCB substrates offer for the development of electronic and biomedical devices.

## 2. Printed Circuit Boards, Parts, Dimensions and Types

**Figure 1:**
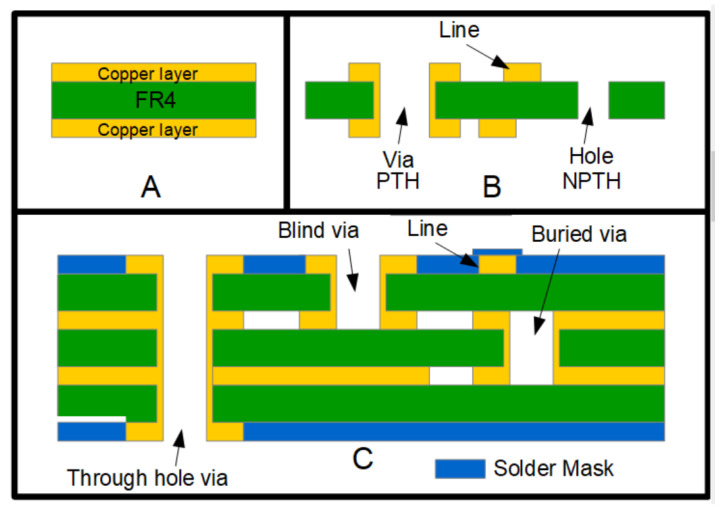
Cross-sectional view of a generic structure of a Printed Circuit Board (PCB). (**A**) Double-side copper layer PCB, where the Flame Retardant 4 (FR4) (green) and the metal (yellow) can be seen. (**B**) Double-side PCB with a copper line, a plated through hole (PTH) via, and a hole (non-plated through hole (NPTH)). (**C**) Four layer PCB with through hole via, blind via, buried via, and a blue solder mask covering the top and bottom layers.

This section summarizes the most important structures of the Printed Circuit Boards, along with their characteristics and dimensions. This section is important to understand the different functionalities that PCBs offer to the user. The generic structure of a Printed Circuit Board (PCB) is shown in Figure 1.

The most common PCB is presented in Figure 1A. As can be seen, it comprises two copper layers (top and bottom) with an intermediate insulation material, which is typically Flame Retardant 4 (FR4). In addition, there is a simpler configuration, with only one copper layer. Figure 1B shows the result of the fabrication process using the double-copper layer, where vias (plated through hole (PTH)) and holes (non-plated through hole (NPTH)) have been fabricated. The vias are characterized as providing electrical and thermal connectivity between the copper layers; in this case, the top and bottom layers.

Figure 1C shows a four-layer PCB. The configuration is similar to the previously noted substrates but includes two additional intermediate copper layers. In this case, the vias can link the four layers, that is, the top, bottom and the intermediate layers. Through vias link the top and bottom layers; blind vias external and intermediate layers; and buried vias link two intermediate layers. The top and bottom surfaces of the final fabricated structure are covered by a blue solder mask. This layer is used to protect the copper lines, releasing only the copper parts used to solder the electronic components. Finally, a silkscreen printing process can be used to name and locate the different components on both the top and bottom surfaces of the PCB. This PCB structure can be used to fabricate more compact electronic circuits. Similarly to this four-layer PCBs, the one- and two-layer PCB can also include solder masks and silkscreen layer.

Apart from these two- and four-layer configurations, the number of total copper layers could be increased. For example, the standard limit that several companies offer is six-layer JLCPCB [16], 14 layers PCBWay [17], e3PCB [18] and PCBgogo [19], and 16 layers Eurocircuits [20] and allPCB [21]. The company Multicircuits boards [22] offers up to eight layers (standard) and up to 28 layers (non standard). Finally, UltimatePCB [23] offers up to 30 copper layers. All these options are for rigid PCB substrates.

In addition, the companies offer the possibility of covering the metal part with tin/lead (hot-air solder leveling (HASL)), gold, silver or Ni/Au/Pd. Regarding the insulating materials, in addition to FR4, different materials can be selected. Furthermore, aluminum is a choice for thermal dissipation applications. Obviously, the aluminum layer is not in contact with the copper layers; a dielectric material is placed between them. Regarding flexible substrates, the materials that could be chosen are polyimide (typical) and polyester (PET) or polyethylene (PE), depending on the company.

Regarding the standard dimensions of the layers, the copper layer can range between 0.5 oz and 13 oz; the insulating layer between 0.17 mm and 7.0 mm; the minimum copper track and spacing 70 μm; minimum diameter vias and hole, 0.15 mm and 0.2 mm, respectively. All these characteristics depend on the manufacturer company. More information can be seen on the manufacturers’ website.

These characteristics are used for electronic circuit interconnections. The next sections show alternative functionalities and uses of the different layers of the Printed Circuit Boards.

## 3. Printed Circuit Boards for Electronics, Sensors and Actuators

As previously noted, the integration of actuators with PCB substrates was performed by Arthur Berry (1913) to fabricate cooking devices. This direct application continues to be interesting, especially for biomedical applications. In addition, thanks to the use of the copper layer, more actuators can be integrated into the PCB, as will be noted below.

### 3.1. Heaters

The integration of heaters and microheaters is mainly based on the Joule effect. Therefore, the copper layer is patterned to fabricate copper lines for heat dissipation. These heaters were integrated in both rigid [24,25,26,27,28] and flexible [29,30,31] substrates for deoxyribonucleic acid (DNA) amplification. The temperature should be as uniform as possible in the area of the reaction chamber. To achieve a constant temperature in an area with a low gradient, another copper layer is used as a plate. The microheaters can be fabricated using the top or bottom layer, or even intermediate layers.

**Figure 2:**
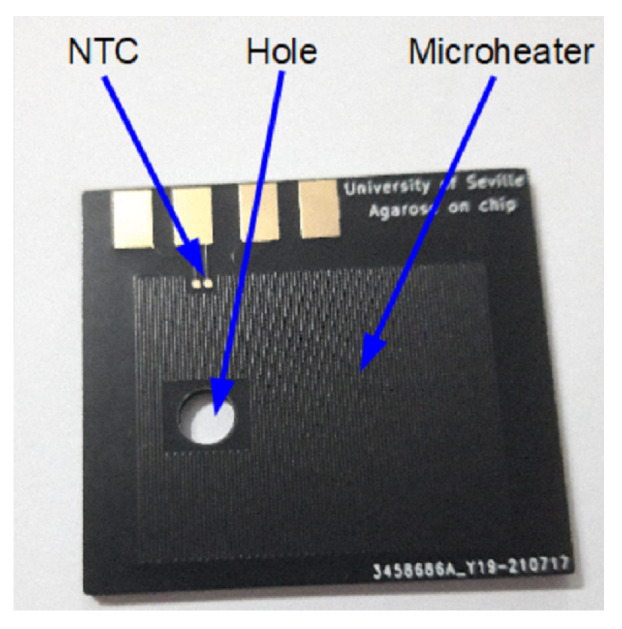
Microheater fabricated using commercially available PCB for agarose gel preparations (reprinted from [32], copyright (2021), Creative Commons License).

For example, the microheaters were used to prepare agarose gel using lab-on-PCB devices [32]. This microheater was fabricated using commercially available PCB substrates, as can be seen in Figure 2. The majority of microheaters are integrated with a thermal sensor to control the temperature set point. In the case of the Figure 2, the sensor is a negative temperature coefficient (NTC) resistor with a surface-mounted device (SMD) package. However, the proper microheater can be used as a temperature sensor [25,28].

In addition, air-flow sensors were developed using PCB-based microheaters [33], as well as devices to study the behaviour of the bubbles [34]. Heaters for the extracellular recording of cardiomyocyte cultures were developed using commercial flexible printed circuit technology [31], and thin copper foil heaters were used to measure the thermal conductivity of polymers [35]. There provide representative examples of microheaters being used for different applications.

### 3.2. Coils

**Figure 3:**
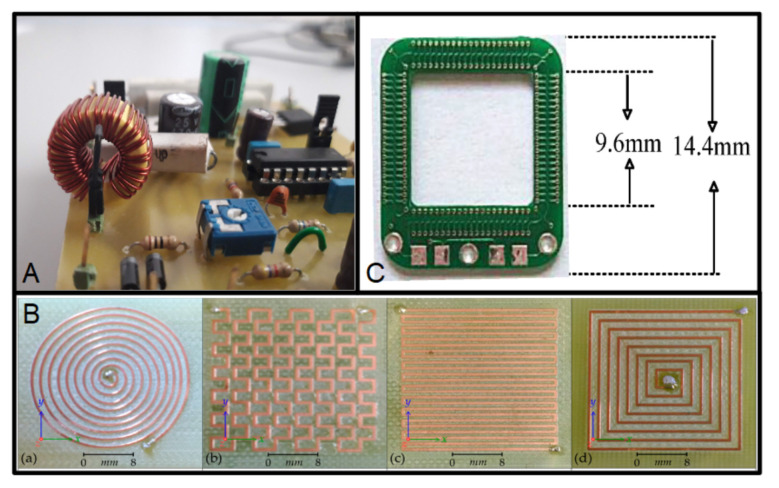
(**A**) Typical inductor assembled to a PCB. (**B**) PCB-based coils with different shapes, (a) Circular planar coil; (b) Mesh planar coil; (c) Meander planar coil and (d) Square planar coil (reprinted from [37], copyright (2018), Creative Commons License). (**C**) Rogowski coil developed on a PCB (reprinted from [38], copyright (2019), Creative Commons License).

The coils are used for developing inductors [36]. The most common inductors are intended to be soldered in a PCB, as in Figure 3A. However, this kind of component can be integrated using the copper layers of a Printed Circuit Board.

The integration of coils on flexible or rigid PCB substrates has been used for wireless power-transmission applications. These coils are fabricated using one copper layer to define the whole structure of the device. The structure is simple: a spiral-shaped copper line in a copper layer, as in Figure 1B, although different topologies are possible [37]. Many devices have been developed using this configuration, for example, printed spiral coils for efficient transcutaneous inductive power transmission [39]. This device is fabricated on a 1-oz copper layer over an FR4 substrate as insulation layer. Similar structures were fabricated for a system with a transmitter and receiver, both of them based on this kind of coil. They are intended for the study of a series of PCB coil matrixes for misalignment-insensitive wireless charging [40]. A current application of these coils is the contactless charger used for handheld devices; for example, smart phones [41]. In addition, the electromagnetic analysis of the alternating current (AC) losses and the practical implementation of PCB planar inductors with a Litz structure were reported [42], as well as the optimization of printed spiral coils for wireless passive sensors [43]. These coils can be fabricated using more than one PCB copper layer; for example, the flow-based electromagnetic-type energy harvester described in [44] included double-sided PCB coils, and the device reported in [45] uses four copper layers two fabricating four coils that are connected in series.

Regarding flexible PCBs, the coils have been used to develop a smartwatch strap wireless power transfer system [46]. To date, the previously noted devices were focused on electronic applications; in contrast, the passive, disposable wireless AC-electroosmotic lab-on-a-PCB, used for particle and fluid manipulation, is an alternative application for biomedical engineering [47].

**Figure 4:**
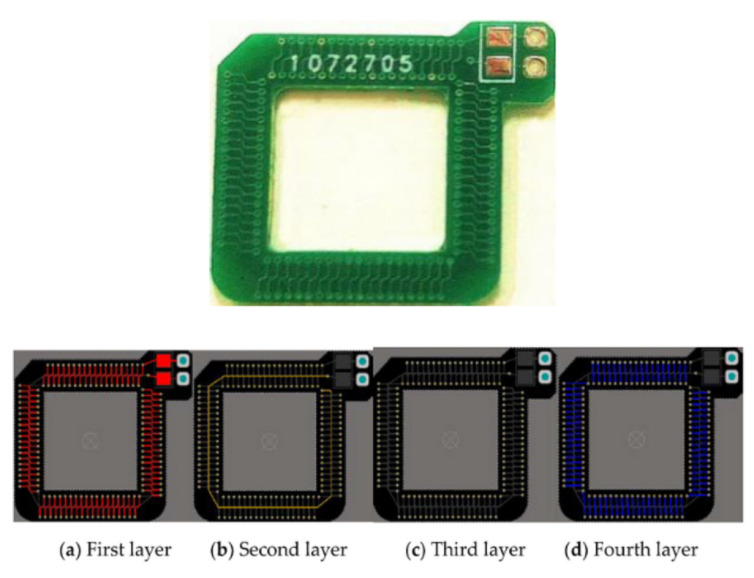
(**Top**) PCB Rogowski coil. (**Bottom**) four-layer board design pattern (reprinted from [51], copyright (2020), Creative Commons License).

A different and widely known coil structure is the Rogowski coils. These are electrical devices used to measure the AC current or high-speed current pulses. Typically, they are helicoidal-based metal lines, with a toroid configuration. The conductor cable is encircled by the toroid to measure the current. Many devices were developed using PCB substrates for power electronic applications as current sensors [48,49,50]. A PCB-base Rogowski coil can be seen in Figure 1C [38]. This kind of coil requires more than one copper layer; for example, the coil reported in [51] is fabricated using four layers, as in Figure 4. This last Rogowski coil is used as a sensor for press-pack insulated gate bipolar transistor chips.

A similar geometry to Rogowski coils has been used for fluxgate sensors [52,53]. This low-cost flat fluxgate magnetic field sensor requires a PCB sandwich structure for both single- and double-core versions.

As previously noted, the coils have also been used for biomedical applications; for example, in devices used for malaria detection. The coils were used to enable the translation of magnetic beads, and the agitation and mixing of those beads within a well. The device is composed of six PCB-based coils on the bottom layer and five coils on the top layer [54]. This device is going to be commented on in the biomedical section, but it is interesting to include in this section to provide a general overview of the PCB-based coil for engineering applications.

### 3.3. Transformers and Motors

The contribution of the copper layer of the Printed Circuit Board to the development of a PCB-based transformer and motor is the definition of coils using copper lines, and an FR4 for electrical isolation.

The most common application of the coils is as transformers. The magnetic components have interesting uses in portable electronic applications, for example, as power modules for handheld computers. As the switching frequency of the converter increases, the size of the magnetic core can be reduced. If the switching frequency is high, that is, a few megahertz, the magnetic core can be avoided. Low-cost, coreless, PCB-based transformers for signal and low-power applications have been proposed [55]. Regarding the use of the core, there are other developments [56,57], but additional materials are required, typically ferrite. In addition, these two last developments use a multiple-PCB structure to fabricate the transformers.

**Figure 5:**
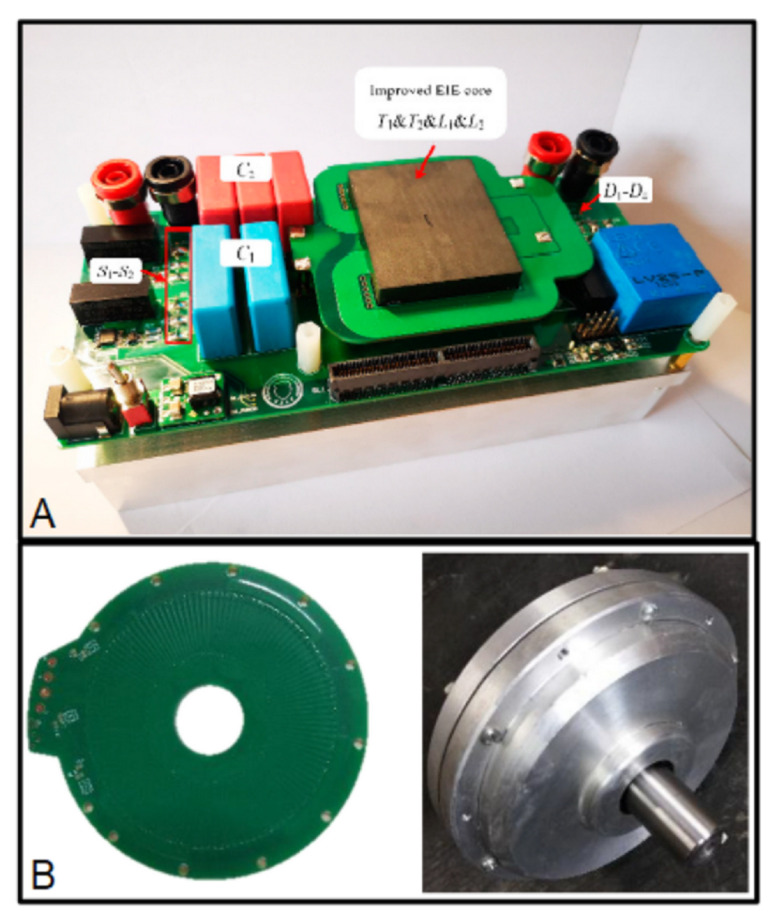
(**A**) PCB-based transformer integrated on a Printed Circuit Board (reprinted from [65], copyright (2020), Creative Commons License). (**B**) Improved PCB stator of a synchronous motor and prototype (reprinted from [67], copyright (2018), Creative Commons License).

The primary and secondary windings are fabricated using the two copper layers of a double-layer PCB [58,59,60,61,62,63], although there are developments using multiple layers (four layers) PCBs [56,64]. In the first case, the FR4 layer offers an electrical isolation ranging between 15 and 40 kV. A PCB-based transformer integrated with a Printed Circuit Board is shown in Figure 5A; in this transformer, the copper windings are fabricated using external and intermediate copper layers, that is, using a multilayer PCB configuration [65]. The transformer reported in [59] uses self-adhesive ferrite polymer composite sheets to shield the magnetic flux from the transformer windings. The work reported in [60] studies the use of PCB-based transformers with windings on opposite sides to achieve the parasitic inductance cancellation of filter capacitors. Investigations into the use of coreless PCB-based transformers for MOSFET/IGBT gate drive circuits are reported in [61]. Finally, a half bridge DC-DC converter for high frequency applications is developed using a step-down power coreless transformer built with a four-layer PCB [64]. The devices are based on a copper coil fabricated using copper layers of the PCB substrate. As can be seen, the contribution of the copper and FR4 layers of PCB—the first one for fabricating the coils and the second one for isolation—make the development of PCB-based transformers possible.

PCB-based motors have attracted increasing interest due to their advantages of a compact size [66], high power density [67], and efficiency [68], design flexibility [69], and low manufacturing costs [70]. Similarly to transformers, PCBs have become a feasible and interesting alternative to conventional round-wire wind. PCB motors can be categorized into two categories depending on the flux direction: radial-flux motors and axial-flux motors [71]. All of them have interesting applications; for instance, PCB-based motors can be used in the development of hard disks [66,68,72]; an axial field permanent magnet motor integrable in the wheel-hub motor of electrical vehicle [73]; for household appliances [70]; for nanosatellites [69] and for a small wind-power system [74].

Multilayer PCBs have been used to fabricate the motors: for example, the PCB stator reported in [67] has 12 layers (Figure 5B); the PCB-based motor for hard disk has six layers in 1-mm-thick PCB, where each layer has nine concentric patterns interconnected by through-holes [68]; a PCB motor intended for use in nanosatellites used a double-layer PCB to integrate the coils [69]; the device reported in [73] requires multilayer PCB (four layers), with 10 PCB-based coils per layer.

Similarly to transformers, the contribution of the copper and FR4 layers of PCB make the development of PCB-based motors possible.

### 3.4. Nanogenerator

The nanogenerators are effective devices for harvesting many kinds of mechanical energy [75,76], especially triboelectric ones, for example, walk energy [77], wave energy [78] or wind power [79], which could be an alternative technology for traditional power generation at large scale. This kind of device can solve the problem of sustainable power and reliable sensing for electronic systems in the Internet-of-Things field. Triboelectric nanogenerators are a promising sustainable power source in self-powered systems.

**Figure 6:**
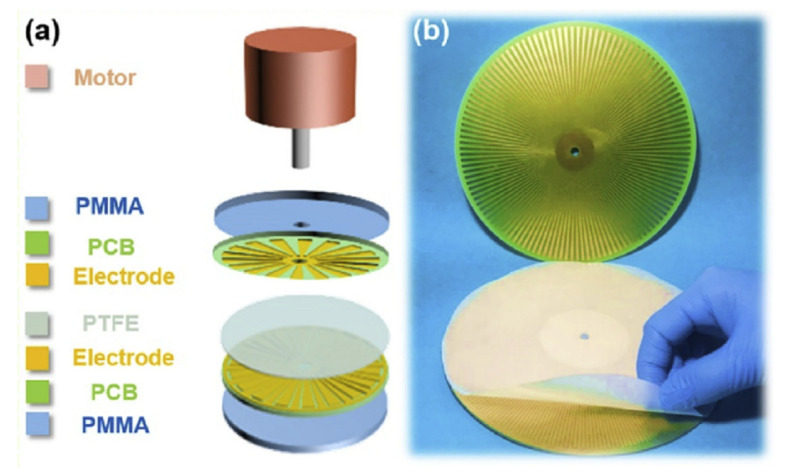
(**a**) Exploded view; (**b**) photograph of a typical rotary disc-shaped triboelectric nanogenerator (reprinted from [83], copyright (2019), with permission from Elsevier).

Printed Circuit Board technology has been used to develop a triboelectric nanogenerator [80,81,82,83,84,85]. These devices are composed of a stator and a rotor, both fabricated using a single-layer PCB. The stator and the rotor have a circular shape, with the FR4 as the substrate, while radial copper electrodes are fabricated in the copper layer. They are radial-arrayed Cu strips with a unit central angle from 10° [81] to 1° [85], depending on the design of the device. An example of the structure can be seen in Figure 6.

These devices are intended to be used in intelligent, self-powered, wireless sensing systems [81], among other uses, where the stator copper electrodes are covered with a polytetrafluoroethylene (PTFE) thin film, and Poly(methyl methacrylate) (PMMA) is adhered to the rotor to strengthen the structure. Another example is a self-powered electrospinning system, developed using PCBs [82]. Similarly to the previous one, the stator is fabricated using FR4 and copper, and uses a polymeric film between the stator and rotor. In this case, the rotor is made of kapton and copper, a flexible Printed Circuit Board. PCB-Based triboelectric nanogenerators have also been used to drive self-powered, on-line ion concentration monitors in water transportation [83], Figure 6. This device is composed of a rigid PCB substrate (FR4 and copper) for both the rotor and stator, with a PTFE film between them. It also uses a PMMA sheet to increase the stiffness of the device.

Triboelectric nanogenerators have been developed for sustainable wastewater treatment via a self-powered electrochemical procedure [85]. In this case, the use of the copper layer is similar to the previously noted ones. Finally, a self-powered smart active radio-frequency identification (RFID) tag was integrated with a triboelectric-electromagnetic nanogenerator [84]. It includes a magnet, two PCB plates covered with copper foils, coated with a solder mask acting as a buffer layer, coils with an aluminum-supporting structure, and a polydimethylsiloxane (PDMS) film between the PCB plates. In this case, there was neither a stator nor a rotor; the working principle is based on approaching and separating the two PCB electrodes.

### 3.5. Fuel Cells

Fuel cells are electrochemical devices, which provide electricity thanks to chemical energy; that is, an electrical current is created using redox reactions. The byproduct of these reactions is water and heat. Several of these devices have been developed, including the proton-exchange membrane fuel cell (PEMFC), alkaline fuel cell (AFC), phosphoric acid fuel cell (PAFC), molten carbonate fuel cell (MCFC), solid oxide fuel cell (SOFC) and direct methanol fuel cell (DMFC) [86]. The main applications of these fuel cells are for power systems [87], cogeneration [88], electric vehicles [89] and portable power systems [90,91].

**Figure 7:**
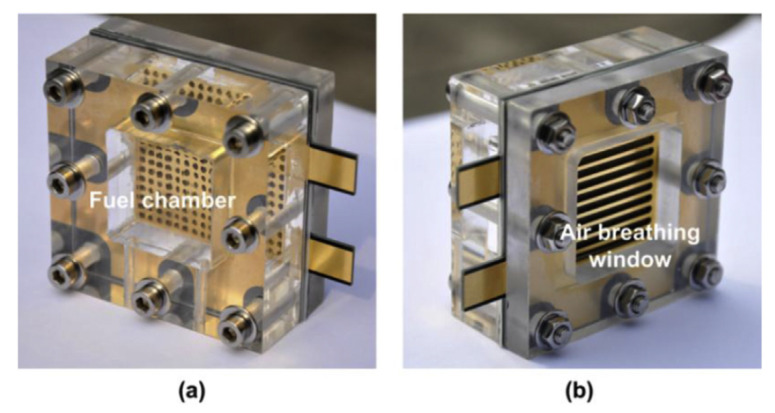
Direct methanol fuel cell: (**a**) fuel chamber with anode; (**b**) air breathing window with the cathode (reprinted from [94], copyright (2015), with permission from Elsevier). As can be seen, the anode and cathode are covered with gold.

Many of these devices have been fabricated using PCBs. Both the copper and substrate (rigid and flexible) layers were chosen to built the PCB version of fuel cells. In general, the main function of the copper layers is the development of the anode and cathode current collector; for example, the devices reported in [92,93,94,95,96] were fabricated using rigid PCBs, Figure 7. The geometry of the anode and cathode openings was studied on [97,98] for PCB devices. In addition, flexible PCBs have been used for fabrication as a current collector [99]. The copper layer corrodes in this kind of device. For that reason, a gold layer covers the copper [100]. This gold layer is an additional material provided by the PCB manufacturer. The gold layers can be seen in Figure 7.

Regarding the microfluidic part of the fuel cells, both copper and FR4 layers were used to fabricate the microchannels for the gas [91,92,96,101]. Similarly to other fluidic devices, the vias were used as inlet and outlet ports. Finally, the required electronic traces can be defined in the same PCB [102].

In summary, the copper, gold and FR4 layers make the development of PCB-based fuel cells possible. The copper layer used to fabricate the anode and cathode current collector and microchannels; the gold layer to avoid corrosion, and FR4 to support the structure and fabricating microchannels. In addition, the vias of the PCB can be used as inlet and outlet ports.

### 3.6. Sensors

A large amount of sensors were developed using Printed Circuit Boards. In this respect, along with the previously commented PCB-based devices, including coils for current and fluxgate sensing, this section describes more functions of the PCB copper layers. In order to do this, the structure of several sensors will be described.

As can be seen, the copper layer of the PCB offers many possibilities to fabricate different devices. This layer continues to be important in the development of sensors.

**Figure 8:**
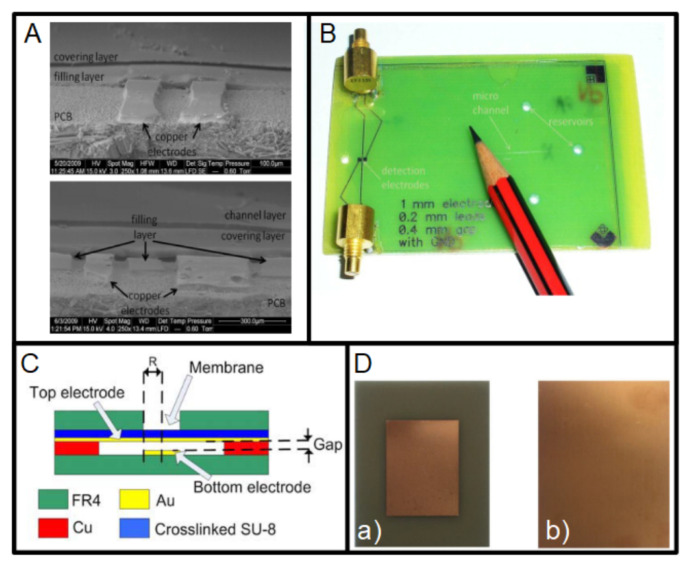
(**A**) Two layers of 30 μm thick dry film photoresist (DFR) laminated on top of electrodes on a PCB (reprinted from [115], copyright (2011), with permission from Elsevier). (**B**) Device with the previously noted electrodes integrated on the PCB (reprinted from [115], copyright (2011), with permission from Elsevier). (**C**) Cross-section view of a pressure sensor with the gap defined using the thickness of the copper layer (copyright (2015) IEEE. Reprinted, with permission, from [104]). (**D**) Sensor fabricated: (a) radiation patch on the upper surface; and (b) metallic ground on the lower surface (reprinted from [111], copyright (2018), Creative Commons License).

Many sensors are based on metal electrodes. Therefore, the copper layer of a PCB is a good choice for their fabrication. In this case, a single-layer PCB is enough. An scanning electron microscope (SEM) image of electrodes fabricated on a PCB substrate can be seen in Figure 8A, and the device on Figure 8B.

The electrodes can be used to fabricate capacitive sensors. For example, the work reported in [103] uses the copper layer to develop the electrodes of a tilt sensor where the dielectric material is silicone oil, and the additional structural material is SU-8 photoresist. The two electrodes were fabricated in the same copper layer. Another interesting application of the copper layer in the fabrication of sensors, in this case, a pressure sensor, is the gap definition [104], Figure 8C. This structure uses the copper layer of 18 μm to define the gap between electrodes. Several values of the copper layer can be chosen, if required for different capacitor gaps.

Moreover, the device reported in [105] is a capacitive sensor array for plantar pressure measurement. The device is composed of two rigid PCBs with a double-sided copper layer, with an electromechanical film technology (EMFIT) electroactive ferroelectric film as a dielectric layer. A different pressure sensor was fabricated using both a conductive flexible film and a rigid PCB [106]. In this sensor, one of the electrodes is fabricated using the copper layer, and the second electrode is the conductive flexible film. Finally, pressure sensors were fabricated using liquid crystal polymer with copper (LCP/Cu) Printed Circuit Boards [107]. This sensor includes a 30-mil substrate for the fixed copper electrode, and a flexible 2-mil film for movable electrode. The electrodes can be fabricated on flexible substrates to develop a deformable sensor; for example, the self-powered sensor for human motion detection and gesture recognition [108].

The vias of the PCB were used as capacitive sensor in the reported work on [109,110]. This device is used to detect gas bubbles inside a fluidic flow, where a tube is inserted in the vias, and the fluid flows are inserted inside the tube. Three electrodes were fabricated using the metal of the via.

The temperature sensors were fabricated using PCBs governed by different working principles. For instance, the wireless temperature sensor reported in [111] is fabricated using a double-sided, copper layer PCB, Figure 8D. In this case, the FR4 layer is chosen as material due to its good properties for microwave and RF applications [112]. The working principle is based on two factors: the metal thermal expansion and the dielectric constant of the FR4 depend on the temperature. Similarly to this sensor, the one reported on [113] uses the copper foil on the polyimide (flexible PCB) as a form of thermal resistance to temperature sensing. A different method for sensing temperature consists of using the temperature dependence of a copper line [25]. In this case, the copper line has two functions: as a microheater and the temperature sensor. Finally, the PCB-based device reported in [114] is used as a multisensor platform to measure temperature, conductivity and pressure.

Conductivity sensors also require electrodes. The conductivity sensor reported in [116] comprised two planar copper electrodes integrated in a Printed Circuit Board. To determine the conductivity, the sensor measures the resistance between the electrodes when they are submerged in an aqueous solution. PCB-based interdigitated electrodes (IDEs) have also been used to detect icing events [117], to measure the concentrations of nitrate [118] or to measure water content in paper pulp [119], etc.

The copper layer can be processed to achieve interdigitated electrodes. These structures can be used as capacitor, as in the LC displacement sensor reported in [120]. This device integrated both a capacitor and a inductor, both built using the copper layers of the PCB. There are many examples of interdigitated electrodes. They will be detailed in Section 4.2.

Another characteristic of the PCB substrate is its easy integration with surface-mounted devices (SMD); for example, two temperature SMD sensors and a SMD heater were integrated in the PCB to develop a flow sensor [121]. Following this idea, an automotive radar sensor was developed [122]. In this system, the electronic control unit and the RF module are realized in standard multilayer FR4 technology using SMD components.

**Figure 9:**
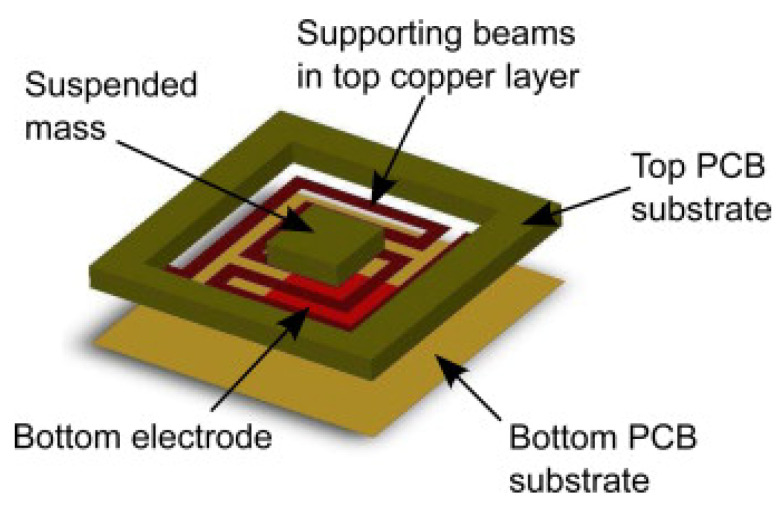
Structure of capacitive PCB-based accelerometer. The beams were fabricated using the copper layer, and the proof mass was defined with the FR4 substrate (reprinted from [124], copyright (2011), with permission from Elsevier).

Printed Circuit Board substrates have also been used to develop accelerometers. The device reported in [123] consists of a metal proof mass, an adhesive tape, and a piece of PCB. The copper layer of the PCB was patterned to fabricate the fixed electrode of the capacitive sensor, and the proof mass was the movable electrode. This device includes the electronic circuit and the sensor in the same PCB substrate. A different device structure was reported in [124], Figure 9. In this case, two rigid Printed Circuit Boards were used to fabricate both the movable and the fixed electrodes. The copper layer of the top movable PCB was used to fabricate the metal plate and the supporting beams. These beams were released by removing the FR4. The FR4over the top metal electrode was not removed in order to define the proof mass and increase the sensitivity. Therefore, the copper layer has two functions: as a metallic electrode and as a movable mechanical structure.

Finally, a similar device structure was reported on [125], where the fixed electrode was fabricated on a rigid PCB substrate using the copper layer, and the top movable electrode was built on a double-sided, copper-layer, flexible PCB.

**Figure 10:**
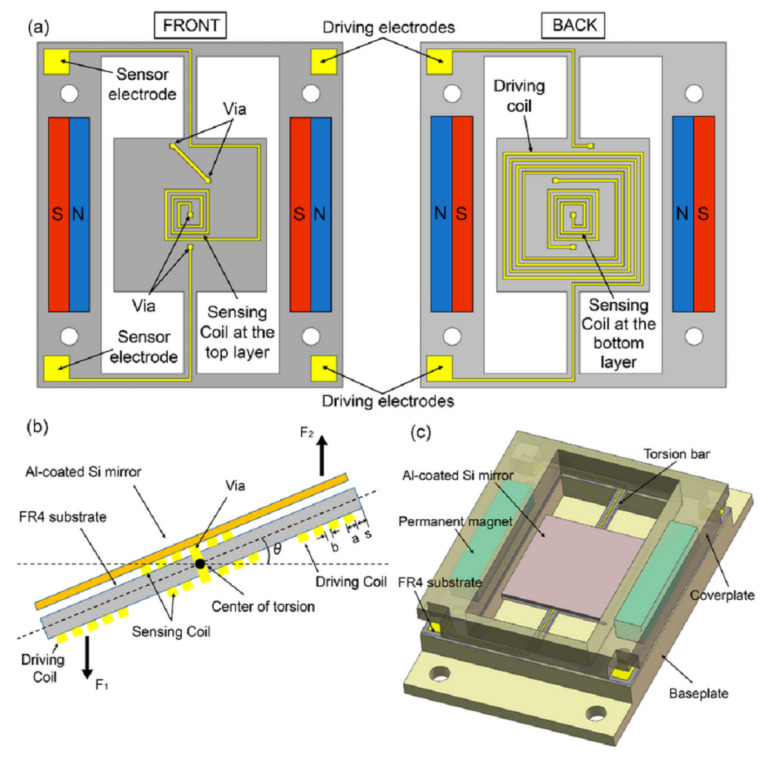
(**a**) Layout of the platform with double-layer copper coils; (**b**) electromagnetic actuation and sensing of the platform with the mirror plate; (**c**) schematic of the assembled scanning micromirror (reprinted from [126], copyright (2018), Creative Commons License).

**Figure 11:**
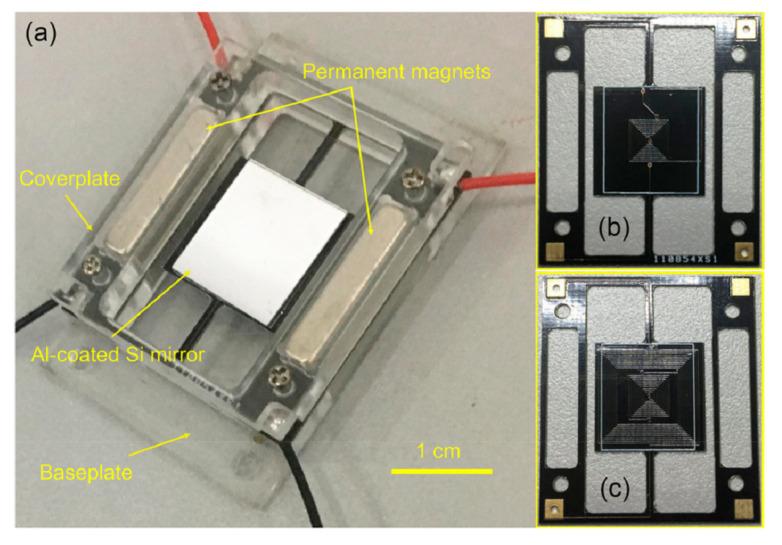
(**a**) Prototype of the electromagnetic scanning micromirror with a plexiglass package; (**b**) front-side view of the platform integrated with copper coils for sensing; (**c**) back-side view of the platform integrated with copper coils for sensing and driving (reprinted from [126], copyright (2018), Creative Commons License).

The FR4 layer was also used to develop a prototype of a electromagnetic scanning micromirror, integrated with an angle sensor [126], as in Figure 10. The angle sensor was fabricated using one copper layer of the PCB, and the driving coil of the micromirror was fabricated on the opposite copper layer of a double-side PCB. The fabricated device is shown in Figure 11.

These devices are examples of sensors where the PCB layers were used to achieve different functions. Moreover, the contribution of the PCB’s layers to this kind of devices include cost-effective characteristics, the sensors’ easy integration with electronic circuits, and commercially available fabrication.

## 4. Printed Circuit Boards for Biomedical Applications

In addition to from the typical use of PCBs as an electronic part biomedical devices, the different layers of the PCBs offer interesting possibilities for the development of many different devices. The biosensors, fluid manipulation devices and lab-on-chip have increased their functionalities and commercialization potential thanks to the use of PCBs.

### 4.1. Fluid Manipulation PCB Devices

Microvalves and micropumps are the most important actuators for fluid manipulation. These devices have been integrated in lab-on-chip devices to control small-volume of fluids, especially for biomedical applications [127,128,129]. Printed Circuit Boards have been used as a substrate to fabricate LoCs due to their differentiating characteristics [130,131,132,133].

**Figure 12:**
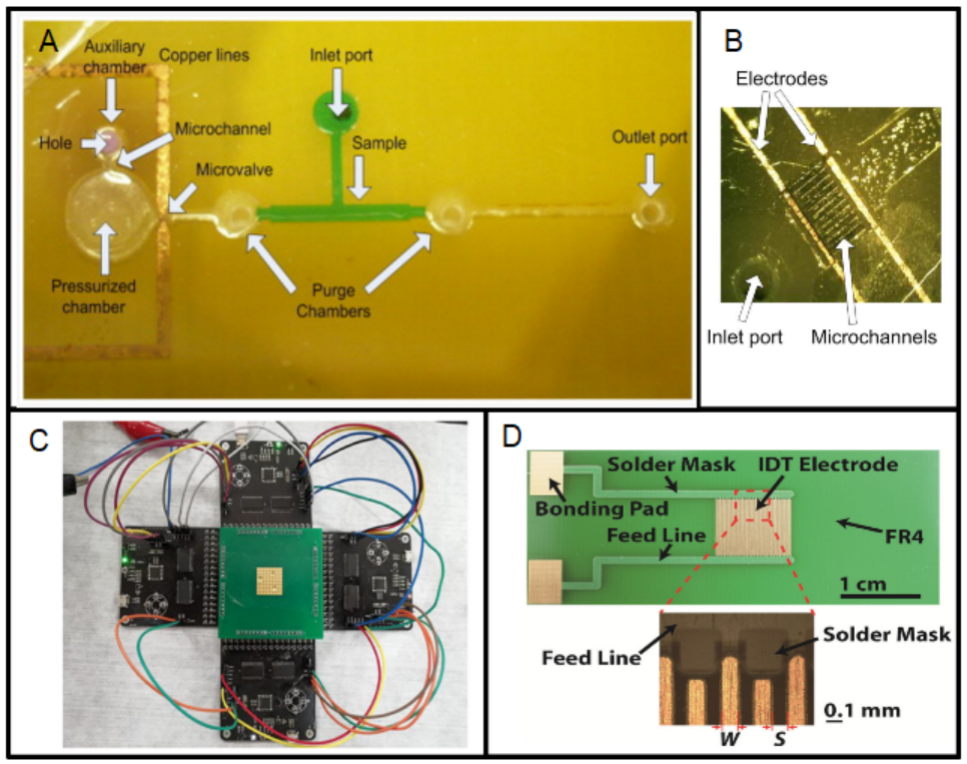
(**A**) Impulsion system based on an SU-8 pressurized chamber and a copper line fuse. (reprinted from [136], copyright (2015), with permission from Elsevier). (**B**) Close view of the electroosmotic part of a PCB-device where the microchannels can be seen (copyright (2013) IEEE. Reprinted, with permission, from [144]). (**C**) Device for fluid manipulation using electrowetting on dielectric on Printed Circuit Board (reprinted from [145], copyright (2020), Creative Commons License). (**D**) Electrochemical PCB-based impulsion chip with detail of the microelectrode fingers (reprinted from [150], copyright (2018), with permission from Elsevier).

The microvalves fabricated using PCBs require an additional material for the microfluidic circuit; for example, SU-8 or PMMA. The microvalves reported in [134,135,136] used a copper line as a fuse to develop a single-use and normally closed microvalve, Figure 12A. This device was fabricated using SU-8 as an additional material, and PCB as a substrate to fabricate both the microvalve and the electrical connection needed for activation. In contrast, a single-use and normally open microvalve was developed in Reference [137]. In this case, the closing was achieved thermally by the melting of a PMMA microchannel using an integrated microheater. PCB-based heaters have also been used for flow driving [134,135,136]. In all these cases, the microheater is a copper line, fabricated using the same procedure as the ones commented on Section 3.1. However, in this case, the function of the final device consists of activating the microvalve, that is, the microheater has a secondary function.

There are microvalves based on PCB, which require a different material to perform flow regulation; for example, the PCB-based microvalves reported in References [138,139,140,141], which use an integrated gold wire as a heater.

The other PCB-based devices for controlling a small volume of fluids are based on electrodes. The fabrication of these electrodes is also based on the typical photolithographic process and etching of the copper layer. Devices for fluid manipulation using electro-osmotic flow [142,143,144] have been developed, as in Figure 12B. The metal electrodes have been integrated in devices based on electrowetting on a dielectric (EWOD). The structure of an EWOD device is shown in Figure 12C [145]. Some interesting applications of these devices are in pyrosequencing or clinical diagnosis [146,147]. In addition, an electrolytic pump for DNA amplification [148,149] has been fabricated. An example of the PCB-base structure is shown in Figure 12D [150]; as can be seen, it is based on IDEs. Finally, a PCB-based surface acoustic wave (SAW) device for particle manipulation was fabricated using IDEs [151]. This kind of device has an interesting application in cell/droplet manipulation [152]. All of these devices use the copper layers to fabricate the electronic lines. As can be seen, these flow-driving devices are intended for use in biomedical engineering. It is worth highlighting that the device reported in Reference [47] integrated a receiving coil for actuation and an AC electro-osmotic micropump based on IDEs in the same flexible PCB substrate. In addition, the system for malaria detection reported in [54] includes a double-layer PCB, with six coils on the bottom layer and five coils on the top layer.

Similarly to electro-osmotic micropumps, the PCB-based copper lines have been used to fabricate electrodes to move charged particles—in this case, through a polymeric gel. This technique is named electrophoresis. It is used for many biomedical applications; for example, in the separation of DNA fragments [153], for clinical diagnosis [154], for rapid, high-resolution DNA sequencing [155], or for the analysis of drugs in biological fluids [156], etc.

Regarding the PCB-based devices used for electrophoresis, those reported in [32] use copper electrodes plated with gold. Adhesive layers were used to cover the copper electrodes in the device reported in [157]. For capillary electrophoresis, a PCB substrate with platinum wires was used to distribute the electrophoresis voltage [158].

Dielectrophoresis is a similar phenomenon, in which a force is exerted on a dielectric particle subjected to a non-uniform electric field [159]. The fabrication of electrodes using the copper layer of the PCB has made the development of biomedical devices possible, for example, devices for cell manipulation [160], and for the suspension of human tumour cells [161]. In addition, the impact of surface roughness on the dielectrophoretically assisted concentration of microorganisms over PCB-based platforms has been studied [162].

Regarding the FR4 layer used for fluid manipulation, the effect of oxygen plasma treatment on the wetting properties of FR4 was investigated [163]. The authors demonstrated that the oxygen plasma treatment of commercially PCB microfluidic structures provides hydrophilic and suitable surfaces for passive microfluidics systems.

Regarding aerodynamic flow control devices, dielectric barrier discharge plasma actuators are typical. This kind of device has been fabricated using Printed Circuit Boards because this decreases the manufacturing cost and weight. Two electrodes are required [164,165,166,167,168,169]: one of them for high-voltage and second one for the ground. These electrodes are fabricated on opposite sides of the PCB substrate, in which the insulation layer is FR4 and the dielectric breakdown voltage is determined by that material, about 50 kV.

Plasma actuator devices are related to biomedical engineering due to the use of plasma for sterilization, ozonization, surface treatments, or skin treatment by plasma for transdermal drug delivery, etc. [169,170]. In these cases, it is interesting to achieve a jet that is perpendicular to the actuator surface. This is possible with PCB-based dielectric barrier discharge plasma actuators, for example, the device reported in Reference [169].

### 4.2. Biosensors and Lab on PCB

Biosensors are very important devices for biomedical applications. These devices have been fabricated using PCB substrates, resulting in devices that can be considered Lab-on-PCBs. In addition, PCB-based biosensors can be integrated into a microfluidic platform to develop Lab-on-PCB systems [132]. The use of PCBs for building small laboratories on chip was proposed in 1996 [171], and the term “Lab-on-PCB” was coined in October 2014 [139] (online version). The first device (1996) was a chemical analysis system (μFIA-system). Since 1996, many devices have been developed. However, the majority of the contributions have been published in the last 5 years.

The review of Lab-on-PCB’s biomedical applications [132] performed a good analysis of the status of this kind of device regarding its applications, and of the role of the PCB (as a sensor and/or electronic reader). The use of PCB layers in biosensors are going to be presented below.

Generally, the biosensors that use PCBs are the electrochemical ones [172]. These devices are based on electrodes. Thus, the copper layer is used to fabricate them. The copper is not a biocompatible material; therefore, a biocompatible cover is needed to protect the biological samples. In addition, this material has to show a stable voltammogram with a wide potential window. This cover is also provided by the PCB manufacturer; for example, a gold layer deposited by electroplating. The work reported in Reference [172] presented a good study of gold as a material for biochemical electrodes on PCB.

**Figure 13:**
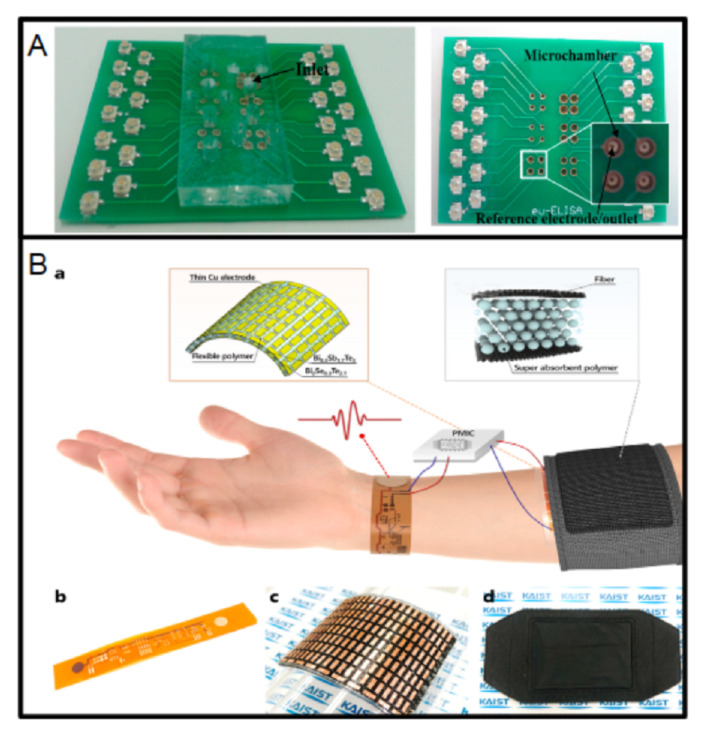
(**A**) Left: Lab-on-PCB integrating microfluidics and PCB microchambers and reference electrodes, right: two-layer PCB before the assembly of microfluidics, comprising microchambers in the top layer and PCB reference electrodes in the bottom layer (reprinted from [174], copyright (2015), Creative Commons License). (**B**) a: schematic diagram of a wearable electrocardiography system, b: flexible electrocardiography module, c: wearable thermoelectric generator, d: polymer-based flexible heat sink (reprinted with permission from [179], copyright (2011), American Chemical Society).

The device reported in Reference [173] is an adhesive and wearable sensor patch used for monitoring sweat electrolytes. It is fabricated using a flexible Printed Circuit Board, where the copper electrodes are covered with Ag/AgCl and paladium. This material (Ag/AgCl) is suitable for developing reference electrodes on PCB [174], as in Figure 13A. This last work demonstrated the good behavior of a pH sensor. In addition, the work reported in Reference [175] also uses silver-based electrodes; in this case, for cancer biomarker detection. Depending on the PCB manufacturer, silver can be ordered as a standard cover. However, the paladium and Ag/AgCl electrodes require additional processes. The HASL cover has also been used to avoid oxidation of the copper, as in the glucose analyzer reported in Reference [176], which is a PCB-based sensor fabricated on a single-side PCB with copper IDEs covered with tin.

The PCB-based chemiresistive carbon dioxide sensor reported in Reference [177] uses silver paste to finish the fabrication of the device. Although this kind of device requires additional processes, it is worth using commercial PCBs to develop them. Many wearable biosensors are based on a flexible printed circuit boards [117,178,179,180,181]; for example, the device reported in Reference [179] can be seen in Figure 13B. In addition, a biosensor for SARS-CoV-2 detection was fabricated using flexible PBCs. In that case, graphene was used as an auxiliary material [182].

Regarding the use of gold in a biomedical PCB-based sensor, flexible Printed Circuit Board electrodes for the high-resolution mapping of gastrointestinal slow wave activity was reported [183]. This PCB includes gold contacts and copper tracks. The Au-plated electrodes were tested as electrodes (working, counter and reference) [184] to develop a glucose biosensor and exploit the covalent immobilization of commercially fabricated PCB-based electrodes. In addition, the characteristics of the Au-coated test strip for blood glucose measurement using PCB electrodes was reported in Reference [185]. Moreover, the devices described in References [186,187] used both gold and silver PCB-based electrodes. Finally, the PCB-based gold electrodes fabricated using the standard ENIG plating process have been applied to the electrochemical sensing of SARS-CoV-2 amplicons and spike protein [188,189]. As can be seen, the copper, gold and silver layers of the PCB are a good choice for developing biosensors.

The PCB substrate can be used as supporting platform for the system. For instance, the work reported in Reference [190], where an impedimetric transducer is wire-bonded to the PCB and then protected using PDMS, and the SAW viscosity sensor with integrated microfluidics, where the sensor is wire-bonded to the PCB as well [191]. Moreover, the electronic circuit and several sensors can be included in the PCB; for example, the PCB-based system reported in Reference [192] includes a pH indicator, conductivity, sodium, and temperature sensors.

The PCB-based electrodes have also been used to develop conductivity measurements [105,116] for biomedical applications. Among others, the capillary electrophoresis device reported in [115] integrates copper electrodes covered with a dry film photoresist; the copper via holes were used as electrodes on the contactless conductivity sensor for bacterial concentration detection [193], and a similar structure was used for a capillary electrophoresis device [194]. Finally, the most-used electrodes are tIDEs. In addition to the ones presented here to show the different functions of the layers, there are many more examples; for instance, an IDE-based PCB biosensor for the measurement of urea [195], an impedimetric biosensor for the detection of lead (II) based on gold-covered IDEs [196], the IDE-based system for electrochemical sensing of nitrite and taste stimuli reported in Reference [197], where the sensor is fabricated using direct laser writing, which showed a good performance for lower concentrations during taste sensing. The capacitive fringing field sensor for moisture measurement used IDEs covered with a solder mask to avoid contact between the electrodes and the liquid [198]. All these IDEs have straight electrodes. However, different configurations are possible; for example, a circular IDE for cell membrane permeabilization procedures [199].

**Figure 14:**
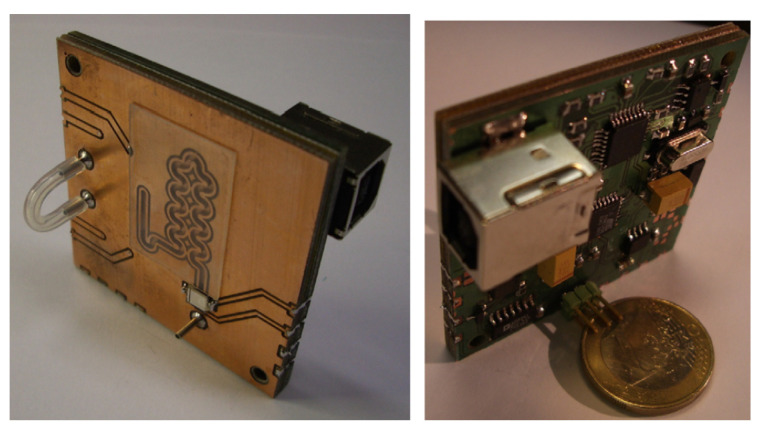
The first lab-on-PCB reported by Stefan Gassmann et al. (reprinted from [11], copyright (2007), with permission from Elsevier).

Lab-on-PCB devices take advantage of every layer in the PCB substrates. Generally, these devices includes sensors, actuators and microfluidics. The microchannels of the microfluidic circuit can be fabricated using the copper lines as walls [10,15]. In addition, in the same work, the authors reported the used of copper lines for the fabrication of microchannels and microheaters; that is, the lines which define the microchannel also define the microheaters. The first lab-on-PCB, including sensors, actuators, electronic components and a microfluidic circuit, was developed for flow injection analysis [11], Figure 14.

**Figure 15:**
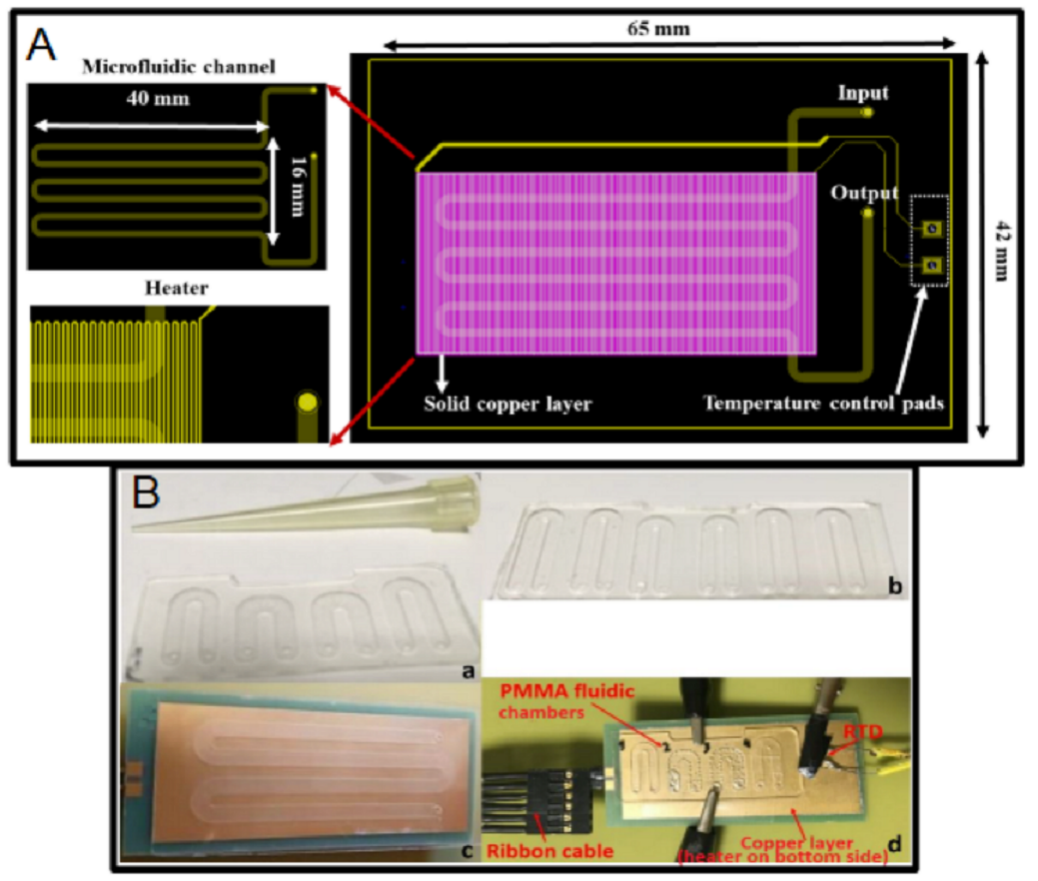
(**A**) Recombinase Polymerase Amplification (RPA)-on-PCB chip design for DNA amplification. The meandering microfluidic channel, the microheater with its electrical pads, and a solid copper layer beneath the microchannel for optimum temperature uniformity are depicted (reprinted from [25], copyright (2021), Creative Commons License). (**B**) a: Poly(methyl methacrylate) PMMA fluidic chip with 4 u-shaped chambers; b: PMMA fluidic with 6 u-shaped chambers; c: PMMA fluidic chip on top of a thin Printed Circuit Board (PCB) microheater with an external temperature-homogenizing copper layer; d: Experimental set-up for temperature measurements during thermocycling of a static micro-polymerase chain reaction (microPCR) chip (reprinted from [24], copyright (2020), Creative Commons License).

The microchannel can also be defined using the solder mask; for example, the Y-channel reported in [200], the microchannels fabricated in [186,187], and the Lab-on-PCB for the isothermal recombinase polymerase amplification of DNA [25]. This last work included a PCB-based microheater, which simultaneously acts as a temperature sensor. The device is fabricated in a four-layer PCB, where the top and bottom copper layers include the contact pads, and the first and second inner layers define a copper plate for temperature uniformity and the microheater, respectively, Figure 15A. The amplification of DNA has also been performed using several Lab-on-PCB devices. The use of flexible PCB has been studied for both continuous-flow and static-chamber configurations [201]. For example, the continuous-flow PCR microdevices [202,203,204], and the static-chamber device reported in Reference [24], Figure 15B. All of them include PCB-based microheaters to define a thermal area to perform the PCR. Finally, the work reported in [205] proposed a structure based on two PCBs. The first one was used to define the microchannels on FR4 by milling, and the second one (multilayer PCB) used integrated microheaters, a copper plate for uniformity, a bottom copper layer for wiring, and a top copper layer for electrodes, which were partially defined by the solder mask.

**Figure 16:**
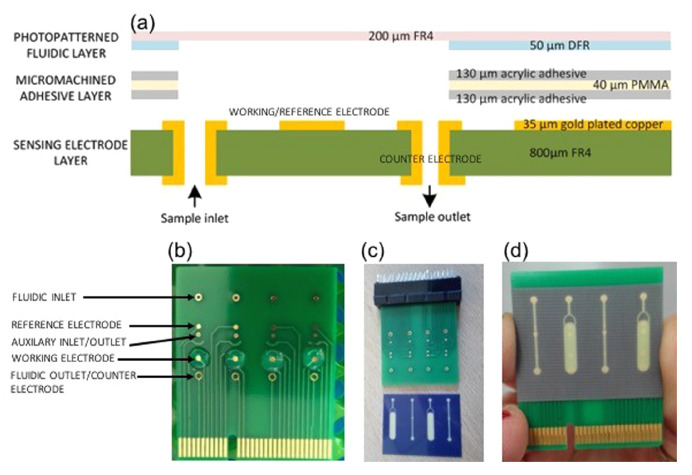
The exploited Lab-on-PCB biosensing platform: (**a**) integrated Lab-on-PCB stack-up; (**b**) Electrochemical Impedance Spectroscopy electrode configuration; (**c**) commercially fabricated PCB biosensing platform; (**d**) sample delivery microfluidics (reprinted from [206], copyright (2019), with permission from Elsevier).

Apart from PCR devices, different devices have been developed. Among others, a peptide-nucleic-acid-based Lab-on-PCB diagnostic platform for DNA quantification [206], Figure 16. This device includes a PCB-based sensing layer which consists of two planar, circular gold-plated electrodes and two cylindrical electrodes, used as the fluidic inlet and outlet using vias.

The “flow injection analysis” device reported in Reference [11] was fabricated using a stack of four PCBs. The top PCB included the microchannel, fabricated using the copper lines of the top side. The rest of the PCB substrates, together with a kapton foil, were used to develop a micropump. In addition, the electronic circuit was included in the bottom PCB. Finally, the detection system consisted of a photodiode soldered on the top PCB.

The microfluidic system for the thermal cycling of seawater samples reported in Reference [207] includes an integrated Peltier cell and a SMD temperature sensor for temperature control, and copper areas fabricated using the top copper layer to make the temperature of those areas uniform. In addition, the device includes the the electronic circuit required for the control. The Lab-on-PCB reported in Reference [32] includes a double-layer PCB for agarose gel preparation and electrophoresis. The top copper layer comprises a PCB-base, gold-plated conductivity sensor based on IDEs, and the pads for electronic connections; the bottom layer includes a PCB-based microheater and SMD temperature sensor to control the temperature.

The flow cytometer reported in Reference [208] comprises copper electrodes covered using a cover glass for the detection and enumeration of circulating tumor cells. Similar structures were reported in References [209,210], where the copper electrodes were covered with SU-8, and [211], where the electrodes were covered using PDMS.

**Figure 17:**
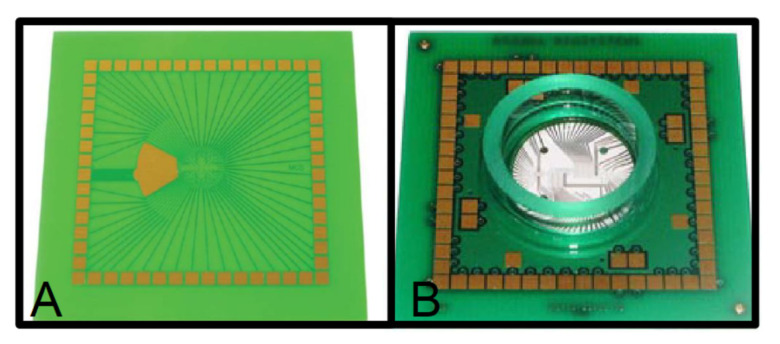
(**A**) Commercial PCB-based microelectrodes arrays of Multichannel Microsystems (model: 60EcoMEA). (**B**) Commercial PCB-based microelectrodes arrays of Ayanda Biosystems™ (model: MEA60 4 × 15 3D).

The solder masks of Printed Circuit Boards are biocompatible materials for cell and organotypic cultures. For example, the company Multichannel Systems (MultiChannel Systems MCS GmbH, Reutlingen, Germany) offer low-cost, PCB-based microelectrode arrays (MEAs) with one copper layer (Eco MEA), and Elpemer^®^2467 or PSR-4000 GP01EU as solder masks. The solder mask is used to isolate the metallic lines, releasing the electrodes of the MEA. The biocompatibility of this MEA has been demonstrated for cardiomyocyte cultures, large slices, or whole-heart preparations [212], Figure 17A. In addition, the biocompatibility of the white solder mask PSR-2000 CD02G/CA-25 CD01 has been checked for a retinal continuous culture system, with good results [213]. Finally, the double-side copper layer PCB has also been used to fabricate MEAs; for example, the one provided by Ayanda Biosystems (Ayanda Biosystems SA, Lausanne, Switzerland) [214]. That MEA included a glass with metal electrodes assembled to the PCB, thanks to the bottom copper layer and the PTH vias, as in Figure 17B.

The use of SMD commercial sensors on the PCB substrates increases the sensitivity of the measurements when their PCB-based counterparts do not provide the required performance. This fact implies an increase in the fabrication complexity, but it is worth if the device fulfills the requirements and works optimally. Among others, the previously noted device includes an SMD temperature sensor [32]; the device reported in reference [215] comprises a silicon-based conductivity sensor and an ion sensitive metal oxide semiconductor (ISFET) pH sensor on the PCB substrate; and the work reported in Reference [190] glued and wire-bonded a transducer array to a Printed Circuit Board.

It is worth mentioning the connection between the portable biomedical devices based on mobile phones or smartphones with Printed Circuit Boards and their layers. For instance, the device used to measure glucose, “HealthPia GlucoPack^TM^ Diabetes Phone”, integrated a blood-glucose-monitoring device into the battery pack of a cell phone [216]. In this case, the biosensor is compatible with the Printed Circuit Boards, especially when using the copper layer to fabricate the electrodes. This biosensor is inserted into the mobile to perform the glucose monitoring. Similarly to this device, the one reported in Reference [217] also needs a biosensor that is compatible with commercial PCB fabrication. The device includes the reader for the biosensor and a Bluetooth module for communication with a smartphone. Regarding the wireless communication between the biosensors and smartphones, a system consisting of a smartphone for gas detection [218] was used to describe an example of an adaption of Near-Field-Communication (NFC) technology to a portable and wireless gas-phase chemical sensing. The authors demonstrated the conversion of inexpensive commercial PCB-based NFC tags into chemical sensors. The device reported in Reference [219] is similar. It includes a planar antenna, an SMD NFC microchip and a connector for electrode interface in the same PCB. It was fabricated by a company (PCBWay, Hangzhou, Zhejiang, China) using a rigid FR4-based as substrate, where the copper layer was used to fabricate the passive components. The device is used for both cyclic voltammetry and chronoamperometry. In addition, the device developed in Reference [220] includes commercial NFC tags with resonant circuits consisting of an integrated circuit chip, a chip capacitor, and resistors and an inductance fabricated using the copper layer, on a flexible PCB with polyethylene terephthalate (PET) substrate. The device is intended for biochemical sensing. Finally, the device reported in Reference [221] is similar to the previous one, and includes a PCB-based system for wireless communication and measurement. In this case, the system is an NFC-enabling, smartphone-based portable amperometric immunosensor for hepatitis B virus detection.

As can be seen, electronic components can be fabricated using the copper layer of the PCB, that is, resistors, capacitors and inductances. These components, together with the possibility of soldering chips on the same PCB substrate, lead the development of very interesting applications, some of which are even compatible with smartphones. In addition, the gold, silver or tin/lead layers, and the solder mask provide the solution to avoiding corrosion and oxidation, and to making the biosensor electrodes functional. All the characteristics that are noted in this section open the possibility of developing attractive and complex applications for biomedical applications.

## 5. Other Uses of PCB Layers

### 5.1. Antennas

Other interesting uses of the PCB layer include the development of antennas. PCB substrate materials are interesting as a dielectric; for example, the TMM3 and RT/duroid 6002 sheets from Rogers are low-loss materials that provide a good high-frequency performance, with excellent electrical and mechanical properties.

Different types of antennas were fabricated using Printed Circuit Boards. The loop antenna is the simplest one. The PCB-based version is a metallic loop fabricated using one copper layer of the PCB [222]. Regarding patch antennas, several approaches have been reported; for example, the one fabricated only one copper layer [223], where the patch and the ground are fabricated on the same side of the PCB; two copper layers have also been used to fabricate these antennas [224]. In this case, the patch and the ground are defined on opposite copper layers of a double-side PCB. Finally, multilayer PCB was used for developing cavity-backed patch antennas [225], where the top layer includes the patch and one ground plane, the intermediate copper layers include ground planes, and the bottom layer is used to fabricate a microstrip copper line to feed the antenna. The PCBs are used to form a cavity to suppress the surface waves. In addition, they can be used to spread the heat. The slot PCB-based antennas uses two copper layers as well. For instance, the antenna reported in Reference [226] has a metal layer for the microstrip line, and the opposite one for the ground, with an E-shaped slot. Meander antennas were fabricated using PCBs [227]. This device is developed using a double-layer PCB, for the meander and ground, and vias are used to connect them. Finally, the inverted F antennas [228] are based on the use of copper layers with similar functionality to the previously presented antennas.

**Figure 18:**
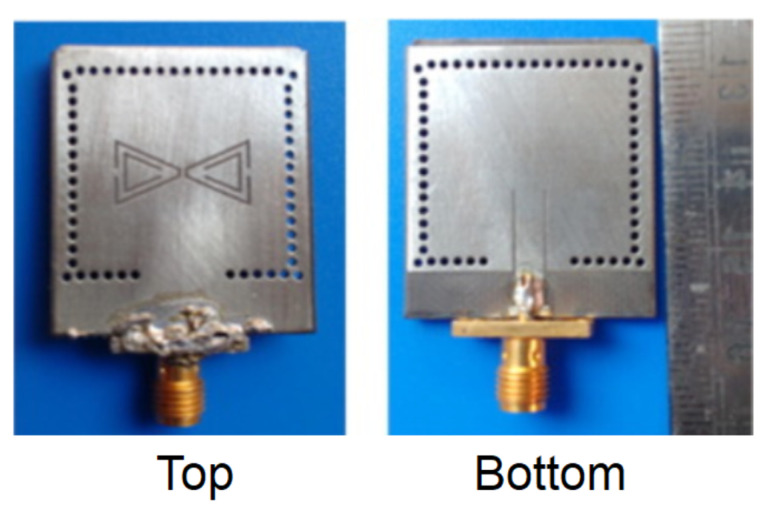
Dual-frequency SIW-based cavity-backed PCB-based antenna. (**Top**): top view where a pair of triangular-complementary-split-ring slots, and vias can be seen; and (**Bottom**): bottom view where the vias can be seen. (reprinted from [229], copyright (2018), with permission from Elsevier).

Figure 18 shows a substrate-integrated, waveguide, cavity-backed slot antenna, fabricated using a PCB substrate. The device includes several metalized vias to avoid energy leakage thanks to the reduction in surface wave propagation. In addition, a pair of triangular-complementary-split-ring slots are included. They are etched on the cavity, which generates a couple of hybrid modes to realize a dual-frequency operation. Although there are no details on the fabrication process, the authors clearly state that they used a single-layered Rogers RT/Duroid 5880 substrate with a thickness of 1.57 mm.

Wearable antennas are based on flexible Printed Circuit Boards [230]. Since the PCBs allow for the integration of different devices, the biosensor described in Reference [173] offers an interesting functionality—that is, it is an adhesive RFID sensor patch, thanks to the integration of an antenna in the PCB.

Regading RF electronics and antennas, IDE structures for electronic filter banks have been developed using a multiayer PCB to assemble the SAW chip [231]. The authors of this work studied the optimal flip-bonding conditions between the piezoelectric wafer and the PCB.

### 5.2. Molds

**Figure 19:**
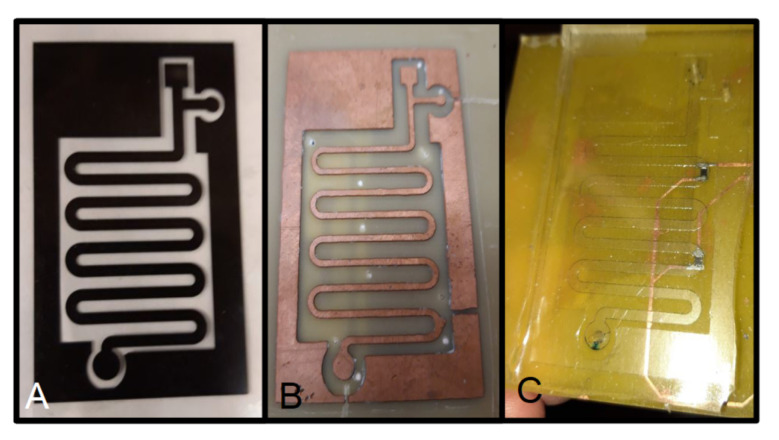
(**A**) Photolitographic mask for fabricating the PCB-based mold. (**B**) Mold for a serpentine microchannel. (**C**) PDMS fabricated device using the mold.

The fabrication of microfluidic devices takes advantage of the use of Printed Circuit Boards. The fabrication of PDMS microfluidic circuit is based on soft lithography; thus, a mold is required. Typically, the molds are fabricated using silicon or SU-8. However, if the dimensional requirements are less demanding, PCB substrates are a good choice. These molds are built using a single-copper-layer PCB [232,233]. In addition, these molds can be used in the hot embossing technique [157,234]. Therefore, thermoplastic materials such as PMMA or polycarbonate can be processed to develop microfluidic devices. Figure 19 shows the photolitographic mask used for fabricating a PCB-based mold, the mold for a serpentine microchannel and the PDMS-fabricated device using that mold.

### 5.3. Flow Focusing

**Figure 20:**
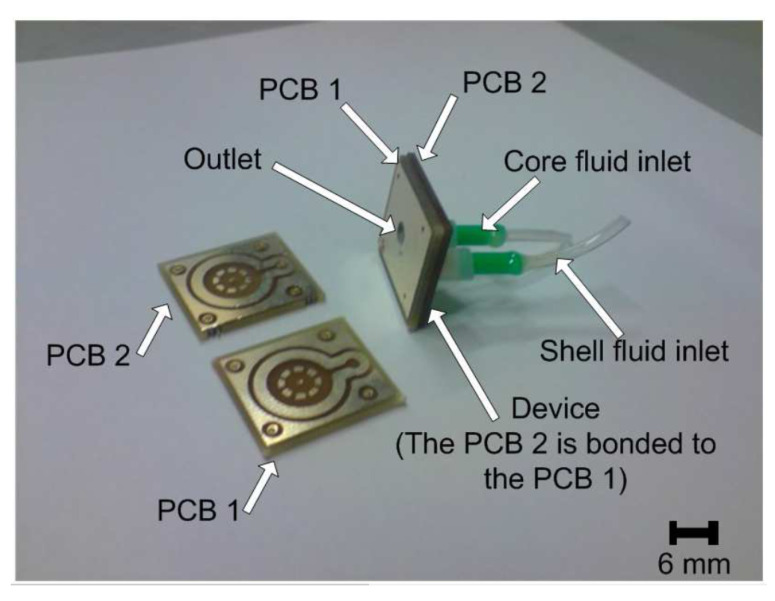
Photograph of the flow-focusing device obtained after the manufacturing process (copyright (2011) IEEE. Reprinted, with permission, from [235]).

As previously noted, the PCB can be used to fabricate microchannels and chambers. The PCB substrates can be used to fabricate flow-focusing devices. A three-dimensional flow-focusing device for microbubble generation was developed [235], as in Figure 20. This device is fabricated using two single-copper-layer PCBs, where the copper lines are used for the microchannels and microchamber, and the vias are used for inserting the core and shell fluids, that is, gas and water, respectively. In addition, a via is used as a microbubbles outlet.

The generation of bidimensional particles only requires microchannels. This kind of device has also been fabricated using PCBs [236]. In this case, the copper layer was used to fabricate those microchannels.

### 5.4. Sacrificial Layer

**Figure 21:**
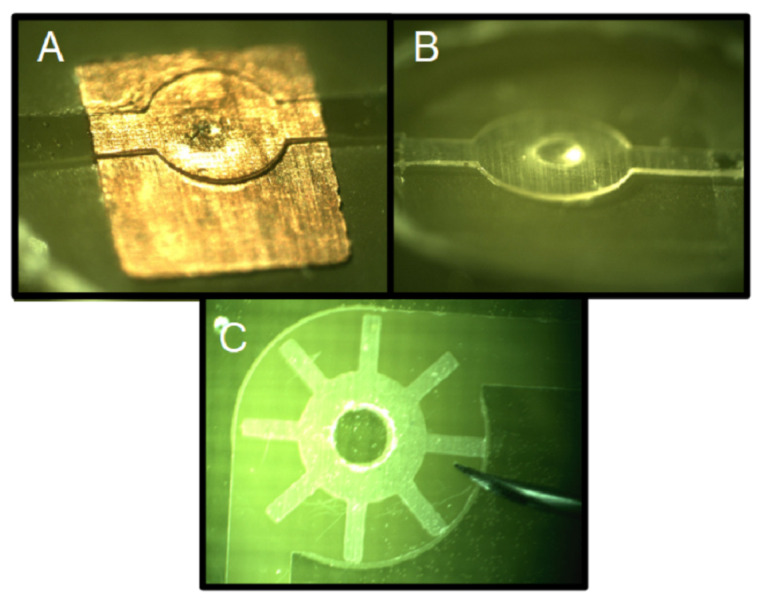
(**A**) Safety valve where the free-standing is not released due to the copper layer (copyright (2010) IEEE. Reprinted, with permission, from [238]). (**B**) Safety valve where the free-standing was released due to the copper layer etching (copyright (2010) IEEE. Reprinted, with permission, from [238]). (**C**) Released wheel for flow measurement made of SU-8 by etching a copper sacrificial layer (copyright (2013) IEEE. Reprinted, with permission, from [240]).

One of the most important steps in MEMS fabrication is based on the use of a sacrificial layer to fabricate free-standing structures. The copper layer of the PCB can be used as a sacrificial layer to fabricate free-standing SU-8 structures [237]. The chemical etching of the copper does not affect the SU-8. For example, the safety valve reported in Reference [238] was fabricated using the copper as a sacrificial layer; Figure 21A shows the system before the copper etching, and Figure 21B shows the final device. The copper layer thickness defines the gap between the free-standing structure and the substrate. This gap can be selected as a function of the available Cu layer thickness offered by the manufacturer. In addition, the copper layer can be used to release SU-8 structures from the PCB substrate [239,240]. Figure 21C shows a released SU-8 wheel for flow measurement, made of SU-8, by etching a sacrificial copper layer.

## 6. Discussion

Thanks to the Printed Circuit Boards, many devices have been fabricated, from purely electronic ones, such as motors or transformers, to biomedical devices, i.e., PCR microdevices. The possibility of integrating different kinds of PCB-based materials led to the fabrication of interesting devices, such as a biosensor with antennas. All these developments are made possible due to the possibilities of all the layers that compose the PCB. The functions of the metallic layers, that is, copper, silver and gold layers, are summarized in Table 1, together with an example. The typical functions of these layers are not included in the Table 1.

The multiple functions of the vias, flexible and rigid substrates are summarized in Table 2, together with an example.

The different metallic layers of the PCBs and their structure make the development of many different devices possible. The layer that offers the most functionalities is the copper layer. The reason for this is that it is a conductive and patternable thin layer. In addition, it is previously deposited when the PCB is bought, with multilayer versions; up to 30 layers are available, depending on the manufacturer. In addition, the vias, the holes and the different materials available for the substrate, mprovide interesting fabrication possibilities. These factors create a technology that can provide devices for different fields, as noted in the paper. It is important to highlight that many devices are fabricated using PCBs. This paper includes representative examples of the devices that take advance of the different fabrication possibilities.

The opportunity to order PCB for a commercial company facilitates its development by researchers and companies, in the same way that foundries offer their services for silicon and glass fabrication for microelectronics and microsystems. Moreover, PCB processing does not require expensive facilities. This fact allows for the development of devices whose fabrication process does not fit the offered by the manufacturer.

This paper presents the PCB functionalities that have been used to date. However, there are options that have never, to the author’s knowledge, been used for a different function than the typical one; for example, aluminum substrate. In this case, the ceramic PCB is used as a supporting structure because it has high mechanical strength and good thermal conductivity [241,242,243,244]. Although there companies offering transparent substrates such as glass or PMMA covered with gold, it would be interesting, from the biological perspective, to include PMMA or polycarbonate as a standard material in the typical fabrication process of Printed Circuit Board manufacturing—that is, a PMMA substrate with copper or gold lines, with vias, holes, solder masks and multilayer versions—while maintaining the quality and the cost. This is the reason for not including transparent Printed Circuit Boards. In fact, there are many developments of these devices, as in References [148,245,246,247].

## 7. Conclusions

Electronic and electrical engineers have used Printed Circuit Boards since the creation of the substrate. Initially, PCBs were used as connection components, and to connect the substrate to different devices, which, in turn, were composed of Printed Circuit Boards. The multiple fabrication options that PCBs offer makes them useful in the development of electronic components, for example, inductors and capacitors. In addition, the possibility of fabricating electrodes opened up the possibility of developing many different sensors and actuators. Moreover, the ability to include several PCB-based devices, such as antennas and sensors, in the same platform, led to the development of many interesting and complex devices.

Many aspects of the biomedical engineering are closely related with electronic engineering; for example, both of them deal with voltages and amperes, such as intracellular and extracellular potential actions, or the management of current in biosensors, among other applications. In addition, the sensors and actuator provided by electronic engineering make the actuation and control of biomedical devices, such as PCR devices and microdevices, possible.

Printed circuit boards have been used in electronic and biomedical engineering, not only for the development of electronic devices, but also in the fabrication of biosensors, actuators and cell/organotypic culture systems.

New devices and new uses for the different layers of the Printed Circuit Boards are being developed. For example, the discovery of new pathogens and diseases leads research into new devices; for example, the Sars-Cov2 virus pandemic led to the development of PCB devices. Finally, all the possibilities that PCB offers, together with its low cost, will lead to the development of potentially marketable devices, the creation of new companies and an improvement in social welfare in the future.

## Figures and Tables

**Table 1:** Metallic layers functions and devices.

PCB Layer	Function	Device (Example)	Ref.
Gold	Biocompatible electrodes	Glucose biosensor	[184]
	Oxidation prevention	Fuel cell	[94]
Silver	Biocompatible electrodes	Cancer biomarker detection	[175]
Tin/Lead	Oxidation prevention	Glucose sensor	[176]
Copper	Microheater	PCR device	[25]
	Uniform temperature plate	DNA amplification device	[25]
	Microchannels	Microfluidic circuit	[10]
	Microfluidic valve	Impulsion system	[134]
	Capacitive electrodes	Tilt sensor	[103]
	Conductivity electrodes	Conductivity sensor	[116]
	Biochemical electrodes base	pH sensor	[174]
	Anode/cathode current collector	Fuel cell	[94]
	Stator/Rotator	Motor	[67]
	Movable electrodes	Accelerometer	[124]
	EWOD electrodes	Pyrosequencing device	[146]
	Electro-osmotic electrodes	Microfluidic device	[144]
	Electrolytic electrodes	Pumping actuator	[150]
	Gap definition	Pressure sensor	[104]
	Temperature sensor	DNA amplification device	[25]
	Mold	Soft Lithography	[232]
		Hot embossing	[234]
	Sacrificial layer	Safety valve	[238]
	Coils/Spiral	Power transmission	[46]
		Current sensor	[48]
		Fluxgate sensor	[52]
		Antenna	[173]

**Table 2:** Vias, solder mask, flexible and rigid substrate functions and devices.

PCB Part	Function	Device (Example)	Ref.
Via	Inlet/Outlet fluidic ports	LoP for DNA quantification	[206]
	Capacitive electrode	Bubble detector	[109]
	Conductivity electrode	Bacterial concentration detector	[193]
	Suppress surface waves	Antenna	[225]
	3D flow-focusing outlet	Bubble generator	[235]
Solder mask	Buffer layer	Nanogenerator	[84]
	Microchannel	Microfluidic circuit	[25]
	Non-contact measurements	Moisture sensor	[198]
	Cell culture	MEA	[212]
	Retina cultures	MEA	[213]
Flex substrate	Flexible electrodes	Pressure sensor	[106]
	Wearable devices	Sweat electrolytes sensor	[117]
Rigid	Supporting structure	Conductivity/pH sensor	[215]
	Microchannels (FR4)	PCR device	[205]
	Hydrophilic surface (FR4)	passive microfluidics	[163]
	Proof mass (FR4)	Accelerometer	[124]
	Structural (FR4)	Micromirror	[126]
	Dielectric layer (Rogers)	Antenna	[229]
	Electrical isolation	Transformer	[61]

## Data Availability

Data is contained within the article.
